# Duplication iléale chez l’adulte révélée par une perforation

**Published:** 2010-05-28

**Authors:** El bouhaddouti Hicham, Ousadden Abdelmalek, Alaoui Lamrani Youssef, Benjelloun Bachir, Kamaoui Imane, Tizniti Siham, Mazaz Khalid, Ait Taleb Khalid

**Affiliations:** 1 Service de chirurgie viscérale, centre hospitalier Hassan II, Fès, Maroc; 2 Service de radiologie, centre hospitalier Hassan II, Fès, Maroc

**Keywords:** Duplication iléale, Kyste, Perforation

## Abstract

Les duplications intestinales sont des malformations digestives rares (0,2 % des malformations de l’enfant). Elles sont diagnostiquées généralement avant l’âge d’un an, mais elles peuvent rester asymptomatiques et diagnostiquées à l’âge adulte. Le diagnostic est fait le plus souvent lors de laparotomie faite en urgence devant une complication. Elles sont traitées par résection chirurgicale.

## Introduction

Les duplications digestives sont des malformations rares, elles représentent moins de 0,2% des malformations de l’enfant [1]. Elles sont tubulaires ou kystiques et siègent sur un segment du tube digestif, de la cavité buccale à l’anus. Elles comportent une paroi à double tunique musculaire tapissée d’une muqueuse de type digestif souvent ectopique (gastrique, pancréatique). Elles peuvent être communicantes ou non avec la lumière intestinale [2]. Ces duplications sont diagnostiquées le plus souvent avant l’âge de 1 an, mais peuvent rester asymptomatiques et ne se révéler qu’à l’âge adulte. Nous rapportons l’observation d’un adolescent qui a présenté une duplication iléale kystique qui s’est manifesté par une perforation.

## Patient et cas clinique

**Figure 1:**
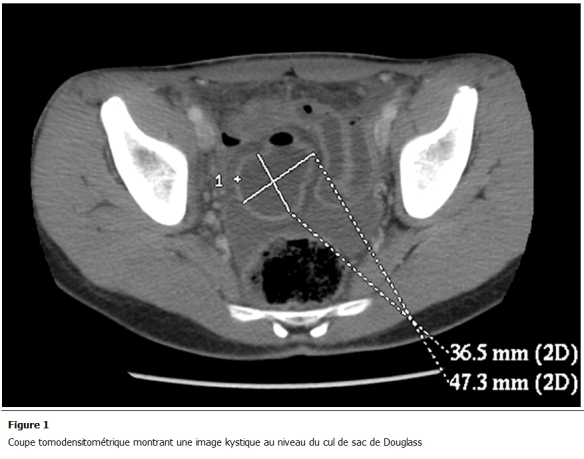
Coupe tomodensitométrique montrant une image kystique au niveau du cul de sac de Douglass

**Figure 2:**
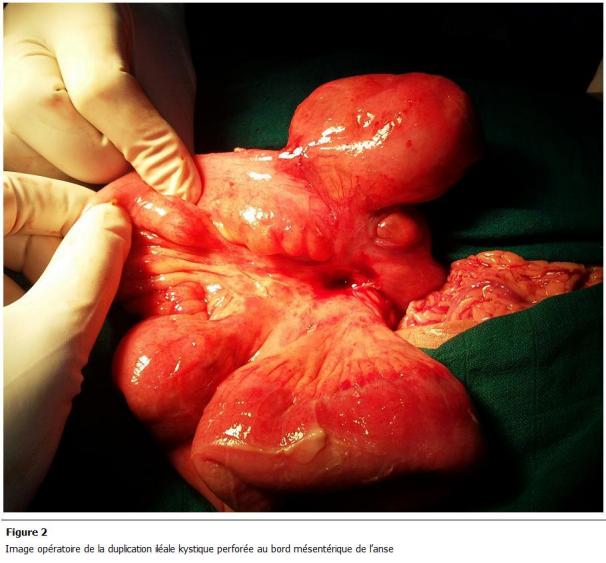
Image opératoire de la duplication iléale kystique perforée au bord mésentérique de l’anse.

**Figure 3:**
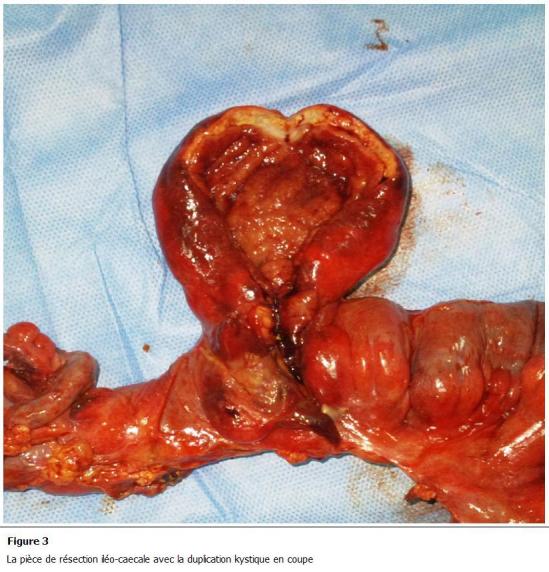
La pièce de résection iléo-caecale avec la duplication kystique en coupe

Patient âgé de 17 ans, sans antécédents médicaux notables, admis aux urgences pour des douleurs abdominales. Ces douleurs ont commencé 2 jours auparavant, de siège sous-ombilical, d’une intensité croissante associées à des vomissements alimentaires sans troubles de transit ni hémorragie digestive extériorisée ni autre signes accompagnateurs. A l’examen, il était fébrile à 38,5 °c avec une défense abdominale sous ombilicale. La radiographie de l’abdomen sans préparation était sans particularité. Le bilan biologique a montré une hyperleucocytose à 18000 élément/mm³. Une tomodensitométrie (TDM) abdominale (Figure 1) a montré une image kystique au niveau du cul de sac de Douglas prenant le contraste en périphérie avec un épanchement intrapéritonéal en inter-anses et dans les gouttières pariéto-coliques. Le patient a été opéré le jour même, par une laparotomie médiane. Il y avait un épanchement purulent avec des fausses membranes. La formation kystique vue au scanner correspondait à un kyste appendu à la dernière anse iléale de 5 cm environ de grand axe avec une paroi qui a l’aspect de la paroi intestinale (Figure 2). Ce kyste était adhérant à une autre anse iléale située à 20 cm en amont. Il était perforé à sa base et il communiquait avec les 2 anses. On a procédé à une résection iléo-caecale emportant les 2 anses et le kyste avec une double stomie iléo-colique à la Bouilly-Volkmann. A l’ouverture de la pièce opératoire (Figure 3), le kyste contenait un liquide noirâtre et avait une muqueuse lisse. Les suites opératoires étaient simples, le patient est sorti de l’hôpital le 5^e^ jour de son hospitalisation. Une chirurgie de rétablissement de la continuité digestive est prévue 3 mois plus tard.

## Discussion

Les duplications intestinales sont rarement découvertes chez l’adulte (4 à 12% des duplications digestives) [3]. Dans une étude américaine portant sur 4 des plus grands centres de chirurgie pédiatrique américains, Mac Pherson a estimé la fréquence de ces duplications à 2 ou 3 cas par an [2].

La duplication iléale (DI) est la plus fréquente des duplications digestives dont elle représente 30 à 60% [2-4]. La forme kystique (retrouvée chez notre patient), représente environ 82% des formes, alors que la forme tubulaire est présente dans les 18% des cas restants [2]. Les DI adhèrent au bord mésentérique du grêle ce qui les distinguent du diverticule de Meckel qui adhère au bord anti-mésentérique [5]. La pathogénie des DI reste très discutée. Plusieurs théories ont été avancées (théorie vasculaire, anomalie de différenciation embryonnaire) /8-10/ sans qu’aucune ne puisse expliquer le polymorphisme topographique, l’association à d’autres malformations ou l’existence d’hétérotopie gastrique ou pancréatique [6, 7]. L’affection peut se révéler par des vomissements (24%), des douleurs abdominales (34%), une masse palpable (10,5%) [8], parfois par une complication révélatrice : syndrome occlusif, hémorragie ou perforation comme pour notre patient [9]. L’échographie et la TDM abdominales peuvent évoquer le diagnostic en présence d’une image kystique intra-péritonéale chez l’enfant. Cependant, dans la majorité des séries rapportées, le diagnostic est fait en per-opératoire [2]. Dans notre cas, la TDM abdominale a permis d’objectiver la lésion kystique sans pouvoir affirmer son origine.

Le traitement de ces affections consiste en une résection iléale emportant la malformation, évitant ainsi la survenue des complications liées aux secrétions acides ou enzymatiques de leur muqueuse hétérotopique [10].

L’anastomose digestive se fera au même temps opératoire s’il n’y a pas de contre indications comme la présence de péritonite. Sinon une stomie sera alors confectionnée (comme pour notre patient) et un rétablissement de la continuité se fera 2 à 3 mois plus tard.

## Conclusion

Les DI sont très rares chez l’adulte car elles sont le plus souvent diagnostiquées chez l’enfant. Elles ont un polymorphisme clinique qui rend leur diagnostic difficile. Elles sont découvertes le plus souvent lors des laparotomies faites pour une complication. Elles sont traitées par résection intestinale emportant la lésion.

## Conflits d’intérêts

Les auteurs ne déclarent aucuns conflits d’intérêts

## Contribution des auteurs

KAT, KM, AO et HB ont opéré le patient. YAL IK et ST ont réalisé les imageries, les ont interprétées et ont traité les images. BB a participé à la relecture de l’article. Tous les auteurs ont lu et approuvé la version finale du manuscrit.

## References

[1] Vanneuville Scheye TG, Dechelotte P, Queroy-Malamenaide C, Aufauvre B (1995). Duplication of the digestive tract in children: Apropos of 12 cases.. Ann Chir..

[2] Macpherson RI (1993). Gastrointestinal tract duplications: clinical, pathologic, etiologic, and radiologic considerations.. Radiographics..

[3] Faucheron JL, Cardin N, Bichard P, Rachidi G, Pasquier D, Letoublon C (1998). Jejunal duplication in adults: Case report.. Ann Chir..

[4] Sebastian JJ, Fuentes J, Boldova I, Garcia S, Cardiel MJ (1996). Giant chylous cyst: An unusual presentation of intestinal duplication.. Hepatogastroenterology..

[5] Ildstad ST, Tollerud DJ, Weiss RG, Ryan DP, McGowan MA, Martin LW (1988). Duplications of the alimentary tract: Clinical characteristics, preferred treatment, and associated malformations.. Ann Surg..

[6] Sapin E, Hélardot P, Bienaymé J, Bargy F Doin (1990). Duplications digestives.. Chir Dig Enfant..

[7] Daudet M, Chappuis JP, Daudet N (1967). Symposium on intestinal duplications.. Ann Chir Infant..

[8] Tanabe ID, DiTomaso A, Pinkas H, Pencev D (1995). Massive GI hemorrhage from an ileal duplication cyst in an adult.. Am J Gastroenterol..

[9] Hoshi K, Ohta M, Kanemura E, Koganei K, Takahashi M, Kito F, Fukushima T (2002). A case of ileal duplication presenting with bloody stools.. J Japan Soc Coloproctol.

[10] Holcomb GW, A Gheissari JA, O´Neill Jr, Shorter NA, Bishop HC (1989). Surgical management of alimentary tract duplications.. Ann Surg..

